# Decentralising scientific publishing: can the blockchain improve science communication?

**DOI:** 10.1590/0074-02760190257

**Published:** 2019-08-19

**Authors:** Flávio Codeço Coelho, Adeilton Brandão

**Affiliations:** 1 Fundação Getúlio Vargas, Escola de Matemática Aplicada, Rio de Janeiro, RJ, Brasil; 2 Fundação Oswaldo Cruz-Fiocruz, Instituto Oswaldo Cruz, Laboratório Interdisciplinar de Pesquisas Médicas, Fiocruz, Rio de Janeiro, RJ, Brasil

**Keywords:** science publishing, peer review, ledger, blockchain

## Abstract

We present a decentralised solution for managing scientific communication, based on distributed ledger technologies, also called blockchains. The proposed system aims to solve incentive problems displayed by traditional systems in scientific communication and publication. A minimal working model is presented, defining roles, processes, and expected results from the novel system. The proposed solution is viable, given the current status of blockchain technology, and should lead to a rethinking of current practices and their consequences for scientific communication.

The current model of scientific publishing is in crisis.^1^ ^,^ ^2^ Even without looking at the numbers, just realising that the two most essential elements of the system, the author and the reviewer, work for free, gives us the justified impression that this is not a stable or sustainable model. To be fair, authors do derive value from publishing: their own reputations and incentives from employers to use this publishing model intensively. However, the price they pay for it seems unreasonably high.

Such an imbalance has led to the current situation, in which a number of competing models have emerged, each with its own strengths and weaknesses. They fall into three main categories: (i) traditional closed access: free to publish but access only by subscription; (ii) “gold” open access: free access to article content, author pays to publish; (iii) delayed open access: published articles accessible only after an embargo period.

Beyond these main models, there are hybrid journals in which authors can choose between models and self-archiving and preprint servers whose models do not aim to replace peer-reviewed publication. For a detailed view of open access terminology, see https://www.plos.org/how-open-is-it.

In this article, we consider that the ideal open access model is based on two main features: (1) no economic barriers to either reading or writing of scientific publications, and (2) reviewers and authors are properly incentivised to play their parts in the system. Since someone must pay the cost of any business model, we propose a blockchain-based system in which everyone is rewarded proportionally to the value they provide.

*A true peer-to-peer system* - In all models of scientific publication, scientists are responsible for content generation (as authors) and quality control (as editors and reviewers). Other professionals are also necessary (e.g., typesetters, copy-editors), and they must be remunerated for their work in a self-regulating market.

To fulfil the two main principles of the ideal scientific publication model, as stated above, a number of other features are essential: (a) cost of entry for both consumer and producer of scientific articles must be either zero or negligible; (b) the regulatory framework of this new system should be decentralised, to prevent the rise of a monopoly or any other form of special interest group that could dominate the system; (c) authorship must be absolutely secure; (d) archiving must be decentralised, to prevent censorship; (e) anonymity and privacy must be possible when required, e.g., for blind reviews; (f) reviewing must be decentralised, both in assigning and delivering reviews, but also in terms of rating by peers.

With these features, the whole system begins to resemble a decentralised autonomous organisation (DAO, Fig. 1), a concept born of the Ethereum blockchain community.^3^

**Fig. 1:**
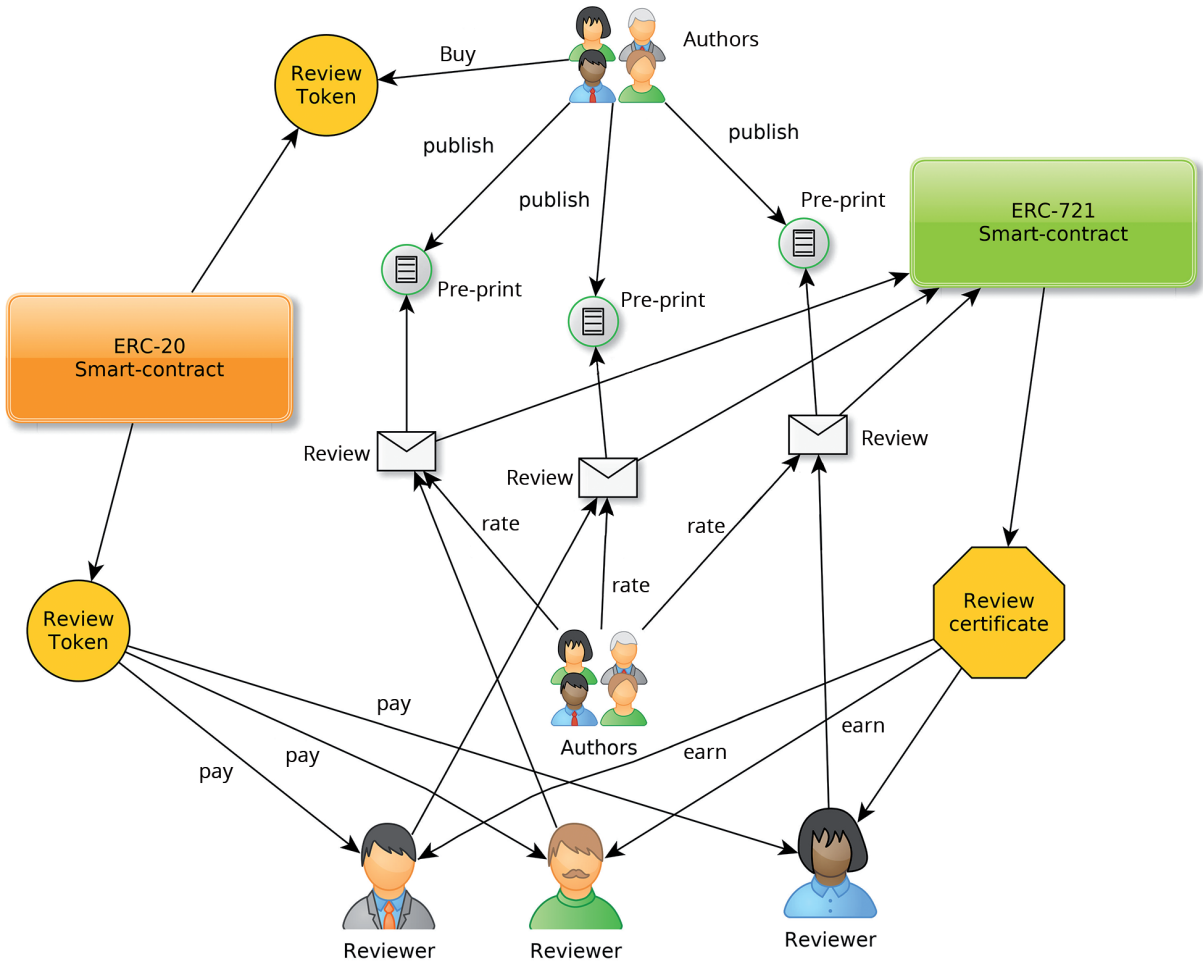
schematic drawing of the proposed decentralised autonomous publishing organisation. Not all elements are included for simplicity

*Scientific publishing as a decentralised autonomous organisation* - For a decentralised organisation to work well, all members of the organisation must have a stake in it. The stake may be represented monetarily, in the form of a token minted by the organisation. Let’s call this token a *Review token* and start by defining the types of members proposed and by looking at their stakes.

*(i) authors* - Authors join the organisation by submitting their preprints. At this point in time, they buy some tokens, which represent the entry cost. Their stake in the organisation is the combined value of their tokens and their peers’ recognition, as measured by article metrics. Authors can be awarded tokens based on the combined metrics of peer-reviewed articles. An award table is defined by the steering committee, to attract high impact research.

*(ii) reviewers* - Reviewers join the organisation spontaneously, incentivised by the rewards of the reviewing process and their own interest in being a part of the organisation. The reviewers’ stake in the organisation is defined by the sum of the tokens they earn from reviewing, plus the average rating of their reviews. The higher their rating, the more likely they are to fetch new review tasks.

*(iii) editors* - Editors begin as reviewers and reach editor status as a result of their reviewer score. They can earn tokens by organising special collections, writing editorials to place publications in a wider context, and other tasks that may help to organise the scientific information flow. In this scenario, editors resemble “curators of science”.

*(iv) To manage the DAO, a steering committee (* *Fig. 2* *) is formed from a random sample of editors and reviewers* - Membership in the committee is kept private, to avoid collusion among members. The mandate duration is also random. The size of this committee must be defined in the DAO smart contract, or can be dynamically set to a fixed fraction of stakeholders.

**Fig. 2:**
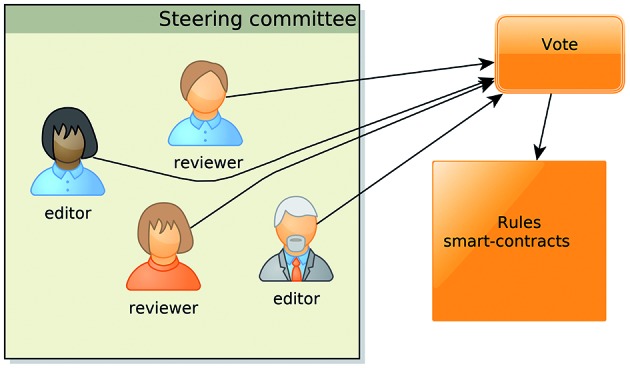
members of the steering committee in a scientific publishing decentralised autonomous organisation (DAO).

*Technology stack* - Throughout this article, we will assume a particular technology stack, which should be understood as one possible implementation. We will use Ethereum as the blockchain platform, and the interplanetary file system (IPFS) as the storage layer. These two elements will be referred to as the *backend system*. Frontend systems will be possible on any web or mobile platform; no particular requirements will be placed on them, other than the assumption that they connect to the backend system through the API specified in the backend system. Examples of frontend systems include: journal websites, institutional repositories, preprint servers, self-publishing scientific platforms (e.g., Zenodo, a type of preprint server but with more features), and any other organisation interested in participating in this open market, either by providing services or by merely publicising its contents.

*Publication workflow* - In this section, we will describe the workflow of the publication process, establishing the foundation for the introduction of the smart contracts that will encode its logic. It should be noted from the start that changes to this workflow are possible and must be decided and specified by the steering committee, but all control of the process must be delegated to the contracts, whose code is open-sourced to make the entire process transparent. The proposed workflow is composed of the following stages or steps:

*Manuscript submission* - The submission process consists of simply depositing the document on IPFS and submitting its address to the submission contract. This action can be performed by any author, without necessarily going through a journal frontend system. The identification of the author will be based on the Ethereum wallet address used to perform the submission transaction but may make use of other blockchain-based identification systems,^4^ ^,^ ^5^ as well as other traditional academic ID services such as ORCID.^6^ Once submitted, the manuscript enters the status of *preprint*. The submission fee should be set by the steering committee. The fee is locked in the contract to pay the minimum number of independent reviewers for each article, as specified by the steering committee.

*Reviewer allocation* - The list of preprints awaiting review can be read directly from the submission contract and exposed in multiple ways through frontend systems. Reviewers already registered in the system may search for interesting work to review on any frontend of their preference. Once reviewers pick a review task, they are given a predetermined amount of time to turn in their review before the task expires. The first reviewers to turn in good reviews (according to the rating scale) will get paid.

*Review process* - Once a reviewer decides to review a manuscript, he or she must prepare the document with his/her comments and upload it to IPFS. A transaction is made to register the review and broadcast it as being available for rating. This cycle may repeat multiple times until the reviewer is satisfied and makes a separate transaction, indicating a substantive improvement of the article in response to issues raised by his/her review. To avoid review spam, payment is delayed until the review receives an *approval* rating by a minimum number of independent reviewers (details below). The reviewer pays the transaction fees related to submitting their review in the hope that if their review receives a good rating, they will be paid more than the fees cost. The author will respond to a review only once the review is approved (has reached the minimum rating), because the contract will release payment only for approved reviews, and only these will count towards the evolution of the preprint to peer-reviewed status. Since it is hoped the manuscript will improve with time, the authors may decide to make a new submission for a new review cycle. All versions of the manuscript during the review must be preserved to allow for auditing of the review process.

*Review rating* - Review rating is done by authors, editors, or other reviewers, and by general readers, with a minimal stake in the system. Given their stake, they should be incentivised to rate reviews strictly on the basis of fairness and contribution to the manuscript, so that the overall quality of the peer-reviewed publications remains high. Furthermore, in this way, unethical reviewers who might otherwise attempt to provide biased reviews to benefit colleagues or trivial reviews just to earn tokens can be weeded out. Reviewers obtain a certificate (ERC-721 token) for every review approved. The rating of the review is recorded in this token, which will count towards their stake in the system.

*Evolution to peer-reviewed status* - Once a preprint undergoes a review cycle leading to its improvement, it evolves to peer-reviewed status. At this point, all payments locked in the contract when the preprint was submitted will have been released to the reviewers. However, this does not mean the article is permanently closed for alterations: a new review cycle may be started if an author (whether or not he or she is provoked by a reader, curator, etc.) decides to change the article. Starting a new review cycle for an article that already has peer-reviewed status causes a versioning event. The peer-reviewed original receives a ‘version 1’ tag, and the new version goes through the new review process (with a different group of reviewers), leading eventually to a peer-reviewed ‘version 2’. This is necessary to preserve the context of any citations that ‘version 1’ may have already attracted.

Once this open market is established, a number of other independent organisations can be set up on top of it. For example, scientific journals can organise special issues, sell editorial services, promote speedier peer-review, etc.

*Final remarks* - The proposed model for a decentralised autonomous organisation to oversee scientific publication is an idea which builds on various experiments in decentralised science, making use of blockchain technology.^7^ ^,^ ^8^ ^,^ ^9^ The present article is simply a high-level description of such a decentralised system.

The implementation of a decentralised publication system as described here, even with the limitations of its current design, can serve as the cornerstone for a revolution in the way scientific communication is currently done. New decentralised processes and communities can build on these foundations and expand into areas that are traditionally closely associated with science: higher education platforms, science dissemination initiatives, and others. The rehabilitation of the scientific publication as a live corpus of knowledge can lead to a model of continual improvements of scientific work, reducing the fragmentation of knowledge and facilitating the coalescence of results into more cohesive and well-supported theories.

The ideas behind this work represent, in a sense, a return to fundamental principles of the scientific method and its culture of openness, in which central authorities have no role in deciding which results should be publicised, and results should stand on their own against valid criticism from peer scientists.

Although the current presentation of the system was conceived in the context of the Ethereum blockchain, other blockchain platforms are currently available and should be considered for its implementation. Current issues of scalability in transaction rates, present in many blockchain platforms, are not so important for this model. Scientific communication is of rather low volume when compared to other transaction-based systems such as digital payments, which are the staple of most blockchain systems. Near real-time transaction confirmation is not a requirement here, since authors and reviewers can comfortably wait a few minutes for a confirmation.
